# 2-Meth­oxy­anilinium trichlorido­stannate(II)

**DOI:** 10.1107/S1600536813005096

**Published:** 2013-03-06

**Authors:** Sahel Karoui, Slaheddine Kamoun, François Michaud

**Affiliations:** a Laboratoire de Génie des Matériaux et Environnement, École Nationale d’Ingénieurs de Sfax, BP 1173, Sfax, Tunisia; b Service commun d’analyse par diffraction des rayons X, Université de Brest, 6 Avenue Victor Le Gorgeu, CS 93837, F-29238 Brest Cedex 3, France

## Abstract

The title compound, (C_7_H_10_NO)[SnCl_3_], is a new compound with non-linear optical (NLO) properties. The structure is pseudocentrosymmetric; the absence of an inversion centre was proved by the Kurtz and Perry method showing a significant second harmonic generation (SHG) signal about ten times lower than that from potassium dihydrogenphosphate. The crystal structure exhibits alternating organic and inorganic layers parallel to the *ab* plane, which are stabilized by inter­molecular N—H⋯Cl inter­actions.

## Related literature

For related structures, see: Zhang *et al.* (2009 ▶). For the effects of substituents on the inter­nal angles of the benzene ring, see: Domenicano & Murray-Rust (1979 ▶). For NLO and SHG, see: Kurtz & Perry (1968 ▶); Kamoun *et al.* (1995 ▶). For ferroelectricity of related compounds, see: Ben Gozlen *et al.* (1994 ▶). For electric and dielectric properties of related compounds, see: Karoui *et al.* (2013 ▶).
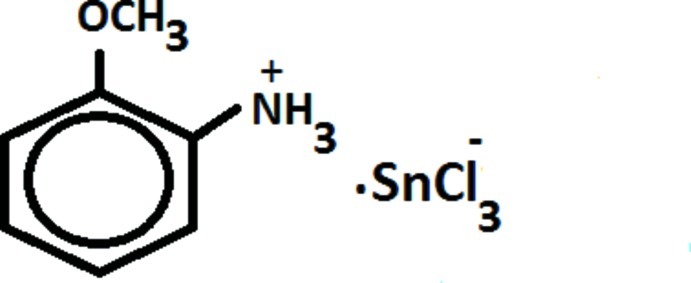


## Experimental

### 

#### Crystal data

(C_7_H_10_NO)[SnCl_3_]
*M* *_r_* = 349.20
Orthorhombic, 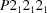

*a* = 7.2030 (2) Å
*b* = 8.3341 (3) Å
*c* = 19.5436 (6) Å
*V* = 1173.21 (6) Å^3^
*Z* = 4
Mo *K*α radiation
μ = 2.82 mm^−1^
*T* = 296 K
0.41 × 0.34 × 0.10 mm

#### Data collection

Agilent Xcalibur (Sapphire2) diffractometer
Absorption correction: multi-scan (*CrysAlis RED*; Agilent, 2012 ▶) *T*_min_ = 0.391, *T*_max_ = 0.765
8969 measured reflections
2392 independent reflections
2236 reflections with *I* > 2σ(*I*)
*R*_int_ = 0.019

#### Refinement

*R*[*F* ^2^ > 2σ(*F* ^2^)] = 0.026
*wR*(*F* ^2^) = 0.064
*S* = 1.14
2392 reflections
120 parameters
H-atom parameters constrained
Δρ_max_ = 0.89 e Å^−3^
Δρ_min_ = −0.69 e Å^−3^
Absolute structure: Flack (1983 ▶), 986 Friedel pairs
Flack parameter: 0.03 (5)

### 

Data collection: *CrysAlis PRO* (Agilent, 2012 ▶); cell refinement: *CrysAlis PRO*; data reduction: *CrysAlis RED* (Agilent, 2012 ▶); program(s) used to solve structure: *SHELXS97* (Sheldrick, 2008 ▶); program(s) used to refine structure: *SHELXL97* (Sheldrick, 2008 ▶); molecular graphics: *DIAMOND* (Brandenburg *et al.*, 1999 ▶) and *Mercury* (Macrae *et al.*, 2006 ▶); software used to prepare material for publication: *WinGX* (Farrugia, 2012 ▶) and *publCIF* (Westrip, 2010 ▶).

## Supplementary Material

Crystal structure: contains datablock(s) I, global. DOI: 10.1107/S1600536813005096/vn2066sup1.cif

Structure factors: contains datablock(s) I. DOI: 10.1107/S1600536813005096/vn2066Isup2.hkl

Supplementary material file. DOI: 10.1107/S1600536813005096/vn2066Isup3.cdx

Additional supplementary materials:  crystallographic information; 3D view; checkCIF report

## Figures and Tables

**Table 1:** Selected bond lengths (Å)

Sn1—Cl1	2.5437 (15)
Sn1—Cl2	2.6489 (11)
Sn1—Cl3	2.5139 (15)

**Table 2:** Hydrogen-bond geometry (Å, °)

*D*—H⋯*A*	*D*—H	H⋯*A*	*D*⋯*A*	*D*—H⋯*A*
N1—H1*C*⋯Cl1^i^	0.89	2.51	3.339 (4)	155
N1—H1*C*⋯Cl3^ii^	0.89	2.85	3.418 (4)	123
N1—H1*A*⋯Cl3^iii^	0.89	2.53	3.329 (4)	151
N1—H1*B*⋯Cl2	0.89	2.54	3.371 (6)	157
N1—H1*B*⋯Cl1	0.89	2.94	3.515 (4)	124

Symmetry codes: (i) 
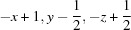
; (ii) 
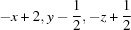
; (iii) 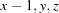.

**Fig. 1.**
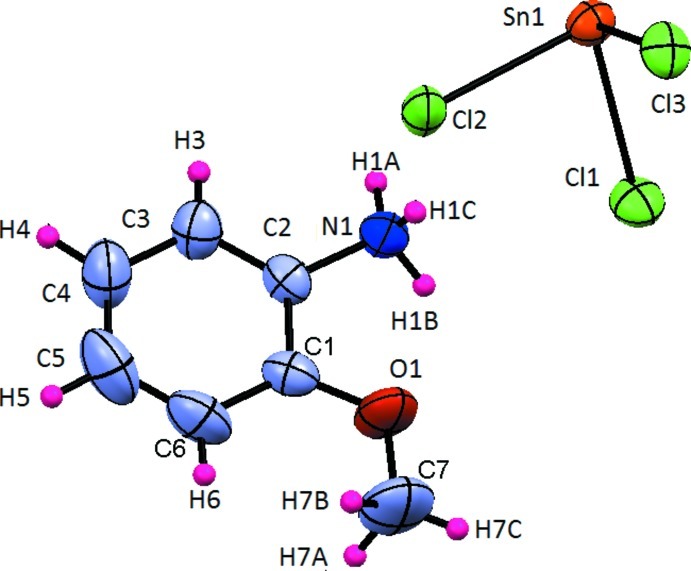
Perspective view of the title compound with displacement ellipsoids drawn at the 50% probability level and H atoms represented by small spheres of arbitrary radii.

**Fig. 2.**
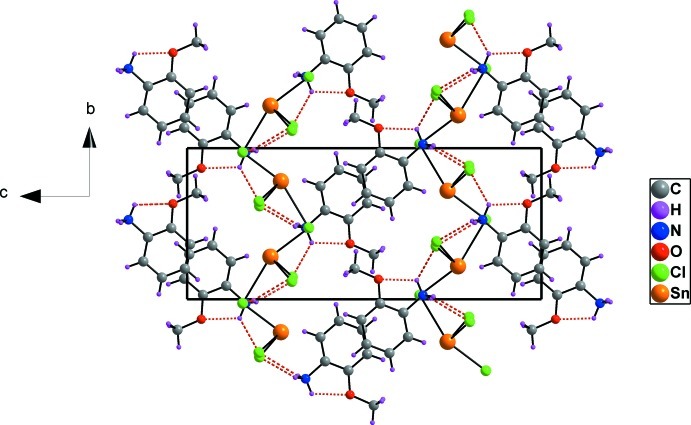
The crystal packing of the title compound viewed along the [100] axis showing the hydrogen bonding network.

## supplementary crystallographic information

### Comment

Considerable attention has been devoted to inorganic-organic hybrid materials over recent years (Zhang *et al.*, 2009). These hybrid materials have interesting physical properties such as ferroelectricity (Ben Gozlen *et al.*, 1994), non-linear optics (Kamoun *et al.*, 1995) as well as electrical conductivity and dielectric relaxation (Karoui *et al.*, 2013). Herein we report the structure of a new non-linear optical material. The structure can be solved and refined in both *P*2_1_2_1_2_1_ and *Pmnb*, the refinement in the latter space group being of less quality than the one in *P*2_1_2_1_2_1. _The NLO response of C_7_H_10_NO.SnCl_3_ has been evaluated by performing SHG on a powder sample using the Kurtz and Perry powder technique (Kurtz & Perry, 1968). The NLO effenciency of C_7_H_10_NO.SnCl_3_ was found to be 10 times lower than KDP. [I^2^^ω^/(I ^ω^)^2^] _C7H10NO.SnCl3_= 0.1[I^2^^ω^/(I ^ω^)^2^]_KDP_, ruling out the possibility of the centrosymmetric space group.

The stereochemical activity of the non-bonding valence electrons on tin (II) in the title compound is evident in the asymmetric coordination environment adopted by this atom (Fig. 1). The primary coordination contacts from tin to the three chlorine atoms constitute the trichloro stannate anion [SnCl_3_]^-^. This anion is pyramidal with Sn—Cl distances of 2.5139 (15) Å, 2.5437 (15) Å, 2.6489 (11) Å (Table 1) and Cl—Sn—Cl bond angles of 93.82 (4), 85.52 (5) and 85.10 (5)°. One longer second contact (3.0075 (11) Å) to chlorine atoms on neighboring [SnCl_3_]^-^ anions complete the fourfold coordinate geometry for tin and give rises to an infinite [SnCl_3_]_n_^n-^ chain along the *b* axis (Fig. 2). Each chain is characterized by a short Sn—Sn bond length of 4.2078 (2) Å and a Sn—Cl—Sn bridge angle of 95.92 (3)°. The benzene ring is practically planar with the greatest deviation from the six-atoms least squares plane being 0.0009 Å. The dihedral angle between two benzene rings of the formula unit is 14°. No stabilization is provided by π-π stacking interactions between the benzene rings of the cations (centroid-centroid distances = 4.362 (4) Å). The torsion angle O1—C1—C2—N1 is 0.2 (8)° indicating that the N1—C2 and C1—O1 groups are in the same plane as the benzene rings. The methoxy group of the organic cation makes an angle of 4(1)° with the plane of the phenyl ring and is in short intramolecular contact with O1 (d_N..O_ =2.621 (5) Å). The bond angles in the phenyl groups deviate significantly from the idealized value of 120° due to the effect of the substituent. In fact, it was established that the angular deformations of phenyl groups can be described as a sum of the effects of the different substituents (Domenicano & Murray-Rust, 1979). The benzene ring is regular with C—C—C angles in agreement with the expected *sp*^2^ hybridation. The major contribution to the cohesion and the stability of this ionic structure comes from intermolecular N—H···Cl hydrogen bond interactions which include five relatively long contacts, with H···Cl and N..Cl distances ranging from 2.510 to 2.938 Å and 3.329 (4) Å to 3.515 (4) Å, respectively (Table 2, Fig.2).

### Experimental

Crystals of (C_7_H_10_NO)[SnCl_3_] were obtained by dissolving 50 mmol of orthoanisidinium chloride and 50 mmol of stannous chloride in HCl (1*M*). Metallic tin was added to the obtained solution to avoid the oxidation of Sn(II) to Sn(IV). This solution was then put in desiccators over CaCl_2_. After several days, yellow parallelipipedic shaped monocrystals of appeared in the solution. They were collected and washed with diethyl ether. The NLO response of the title compound was measured as follows. A 1064 nm fundamental laser beam emitted by Q-switched Nd^3+^: YAG nanosecond laser (SAGA from Thales Laser) at a 10 Hz repetition rate and a Schott RG 1000 filter were used. The intensity of the incident beam was varied using a half-wave plate rotated between two crossed polarizers. The laser beam was directed onto both samples (KDP: KH_2_PO_4_ used as reference and C_7_H_10_NO.SnCl_3_) oriented at 45° incidence angle relative to the laser beam. The second harmonic signal at 532 nm was collected from the face of the sample at 90° compared with the incident beam. The variation of the second harmonic intensity scattered from KDP or C_7_H_10_NO.SnCl_3_ was recorded as a function of the second harmonic reference signal provided by NNP (N-4 nitrophenyl –prolinol) a high NLO material.

### Refinement

After introducing anisotropic thermal factors for the non hydrogen atoms and isotropic ones for H-atoms, the hydrogen atoms were localized and optimized to fixed positions; their contributions were isotropically introduced in the calculations but not refined. The H atoms bonded to the C and the N atoms were positioned geometrically (the C—H and N—H bonds were respectively fixed at 0.96 and 0.89), and allowed to ride on their parent atoms.

### Crystal data

**Table:**

(C_7_H_10_NO)[SnCl_3_]	Cell parameters from 8969 reflections
*M**_r_* = 349.20	*D*_x_ = 1.977 Mg m^−^^3^*D*_m_ = 2.010 Mg m^−^^3^*D*_m_ measured by Flotation
Orthorhombic, *P*2_1_2_1_2_1_	Melting point: 413 K
Hall symbol: P 2ac 2ab	Mo *K*α radiation, λ = 0.71073 Å
*a* = 7.2030 (2) Å	Cell parameters from 8969 reflections
*b* = 8.3341 (3) Å	θ = 2.1–27.0°
*c* = 19.5436 (6) Å	µ = 2.82 mm^−^^1^
*V* = 1173.21 (6) Å^3^	*T* = 296 K
*Z* = 4	Square, yellow
*F*(000) = 672	0.41 × 0.34 × 0.10 mm

### Data collection

**Table:**

Agilent Xcalibur (Sapphire2) diffractometer	2392 independent reflections
Radiation source: Enhance (Mo) X-ray Source	2236 reflections with *I* > 2σ(*I*)
Graphite monochromator	*R*_int_ = 0.019
Detector resolution: 8.3622 pixels mm^-1^	θ_max_ = 26.4°, θ_min_ = 3.0°
ω scans	*h* = −8→9
Absorption correction: multi-scan (*CrysAlis RED*; Agilent, 2012)	*k* = −10→9
*T*_min_ = 0.391, *T*_max_ = 0.765	*l* = −24→24
8969 measured reflections	

### Refinement

**Table:**

Refinement on *F*^2^	Secondary atom site location: difference Fourier map
Least-squares matrix: full	Hydrogen site location: inferred from neighbouring sites
*R*[*F*^2^ > 2σ(*F*^2^)] = 0.026	H-atom parameters constrained
*wR*(*F*^2^) = 0.064	*w* = 1/[σ^2^(*F*_o_^2^) + (0.0195*P*)^2^ + 2.2881*P*] where *P* = (*F*_o_^2^ + 2*F*_c_^2^)/3
*S* = 1.14	(Δ/σ)_max_ = 0.001
2392 reflections	Δρ_max_ = 0.89 e Å^−^^3^
120 parameters	Δρ_min_ = −0.69 e Å^−^^3^
0 restraints	Absolute structure: Flack (1983), 986 Friedel pairs
0 constraints	Flack parameter: 0.03 (5)
Primary atom site location: structure-invariant direct methods	

### Special details

**Table:**

Geometry. All esds (except the esd in the dihedral angle between two l.s. planes) are estimated using the full covariance matrix. The cell esds are taken into account individually in the estimation of esds in distances, angles and torsion angles; correlations between esds in cell parameters are only used when they are defined by crystal symmetry. An approximate (isotropic) treatment of cell esds is used for estimating esds involving l.s. planes.
Refinement. Refinement of *F*^2^ against ALL reflections. The weighted *R*-factor *wR* and goodness of fit *S* are based on *F*^2^, conventional *R*-factors *R* are based on *F*, with *F* set to zero for negative *F*^2^. The threshold expression of *F*^2^ > σ(*F*^2^) is used only for calculating *R*-factors(gt) *etc*. and is not relevant to the choice of reflections for refinement. *R*-factors based on *F*^2^ are statistically about twice as large as those based on *F*, and *R*- factors based on ALL data will be even larger.

### Fractional atomic coordinates and isotropic or equivalent isotropic displacement parameters (Å^2^)

**Table:**

	*x*	*y*	*z*	*U*_iso_*/*U*_eq_	
Sn1	0.99329 (6)	0.71816 (4)	0.264736 (15)	0.03985 (10)	
Cl1	0.74503 (19)	0.88251 (18)	0.20427 (10)	0.0480 (4)	
Cl2	0.9729 (2)	0.52707 (13)	0.15669 (5)	0.0476 (3)	
Cl3	1.2567 (2)	0.85165 (18)	0.20246 (10)	0.0462 (3)	
C1	0.4660 (8)	0.4713 (7)	0.0448 (2)	0.0460 (13)	
C2	0.4807 (8)	0.4108 (5)	0.1105 (2)	0.0391 (10)	
C3	0.4722 (9)	0.2504 (6)	0.1238 (3)	0.0514 (13)	
H3	0.4823	0.2135	0.1686	0.062*	
C4	0.4489 (10)	0.1437 (8)	0.0712 (3)	0.068 (2)	
H4	0.4431	0.0339	0.0795	0.082*	
C5	0.4343 (11)	0.2026 (10)	0.0061 (4)	0.084 (2)	
H5	0.4185	0.1308	−0.0298	0.101*	
C6	0.4420 (10)	0.3637 (9)	−0.0081 (3)	0.070 (2)	
H6	0.4312	0.3999	−0.0529	0.084*	
C7	0.4756 (17)	0.7027 (9)	−0.0271 (3)	0.099 (3)	
H7A	0.4865	0.8172	−0.0234	0.148*	
H7B	0.3612	0.6763	−0.0496	0.148*	
H7C	0.5779	0.6615	−0.0533	0.148*	
N1	0.5056 (8)	0.5263 (4)	0.16573 (16)	0.0447 (8)	
H1A	0.4333	0.6112	0.1584	0.067*	
H1B	0.6237	0.5574	0.1672	0.067*	
H1C	0.4749	0.4809	0.2054	0.067*	
O1	0.4774 (9)	0.6327 (5)	0.03995 (17)	0.0666 (12)	

### Atomic displacement parameters (Å^2^)

**Table:**

	*U*^11^	*U*^22^	*U*^33^	*U*^12^	*U*^13^	*U*^23^
Sn1	0.03921 (16)	0.03849 (15)	0.04186 (16)	−0.00094 (18)	0.0010 (2)	0.00600 (12)
Cl1	0.0361 (6)	0.0544 (8)	0.0536 (9)	0.0062 (6)	−0.0035 (6)	−0.0039 (8)
Cl2	0.0745 (10)	0.0346 (5)	0.0337 (5)	−0.0027 (7)	0.0002 (7)	0.0003 (4)
Cl3	0.0350 (6)	0.0483 (7)	0.0551 (9)	−0.0056 (6)	0.0058 (6)	−0.0033 (7)
C1	0.042 (4)	0.060 (3)	0.036 (2)	0.003 (3)	−0.002 (2)	0.000 (2)
C2	0.035 (3)	0.048 (2)	0.034 (2)	−0.001 (3)	0.006 (2)	−0.0062 (17)
C3	0.057 (4)	0.046 (3)	0.051 (3)	−0.002 (3)	0.010 (3)	−0.003 (2)
C4	0.086 (6)	0.053 (3)	0.067 (4)	−0.011 (3)	0.009 (3)	−0.015 (3)
C5	0.100 (6)	0.093 (6)	0.060 (4)	−0.016 (4)	0.001 (4)	−0.036 (4)
C6	0.083 (5)	0.090 (5)	0.036 (3)	−0.004 (4)	−0.007 (3)	−0.009 (3)
C7	0.151 (8)	0.094 (5)	0.052 (3)	0.018 (8)	−0.002 (6)	0.031 (3)
N1	0.060 (2)	0.0428 (19)	0.0314 (17)	0.004 (3)	0.000 (3)	0.0015 (14)
O1	0.100 (4)	0.061 (2)	0.0394 (18)	0.012 (3)	0.003 (3)	0.0159 (16)

### Geometric parameters (Å, º)

**Table:**

Sn1—Cl1	2.5437 (15)	C4—H4	0.9300
Sn1—Cl2	2.6489 (11)	C5—C6	1.372 (10)
Sn1—Cl3	2.5139 (15)	C5—H5	0.9300
C1—O1	1.351 (6)	C6—H6	0.9300
C1—C6	1.379 (8)	C7—O1	1.435 (6)
C1—C2	1.384 (6)	C7—H7A	0.9600
C2—C3	1.363 (6)	C7—H7B	0.9600
C2—N1	1.458 (5)	C7—H7C	0.9600
C3—C4	1.370 (8)	N1—H1A	0.8900
C3—H3	0.9300	N1—H1B	0.8900
C4—C5	1.367 (10)	N1—H1C	0.8900
			
Cl3—Sn1—Cl1	93.87 (4)	C6—C5—H5	118.8
Cl3—Sn1—Cl2	85.52 (5)	C5—C6—C1	119.4 (6)
Cl1—Sn1—Cl2	85.10 (5)	C5—C6—H6	120.3
O1—C1—C6	127.1 (5)	C1—C6—H6	120.3
O1—C1—C2	115.0 (4)	O1—C7—H7A	109.5
C6—C1—C2	117.9 (5)	O1—C7—H7B	109.5
C3—C2—C1	122.1 (5)	H7A—C7—H7B	109.5
C3—C2—N1	120.7 (4)	O1—C7—H7C	109.5
C1—C2—N1	117.2 (4)	H7A—C7—H7C	109.5
C2—C3—C4	119.9 (5)	H7B—C7—H7C	109.5
C2—C3—H3	120.1	C2—N1—H1A	109.5
C4—C3—H3	120.1	C2—N1—H1B	109.5
C5—C4—C3	118.3 (6)	H1A—N1—H1B	109.5
C5—C4—H4	120.8	C2—N1—H1C	109.5
C3—C4—H4	120.8	H1A—N1—H1C	109.5
C4—C5—C6	122.4 (6)	H1B—N1—H1C	109.5
C4—C5—H5	118.8	C1—O1—C7	117.9 (5)
			
O1—C1—C2—C3	−179.9 (6)	C3—C4—C5—C6	0.0 (12)
C6—C1—C2—C3	0.0 (9)	C4—C5—C6—C1	0.2 (12)
O1—C1—C2—N1	0.2 (8)	O1—C1—C6—C5	179.7 (7)
C6—C1—C2—N1	−179.8 (5)	C2—C1—C6—C5	−0.2 (10)
C1—C2—C3—C4	0.2 (10)	C6—C1—O1—C7	−4.0 (12)
N1—C2—C3—C4	−180.0 (6)	C2—C1—O1—C7	176.0 (7)
C2—C3—C4—C5	−0.1 (10)		

### Hydrogen-bond geometry (Å, º)

**Table:**

*D*—H···*A*	*D*—H	H···*A*	*D*···*A*	*D*—H···*A*
N1—H1*C*···Cl1^i^	0.89	2.51	3.339 (4)	155
N1—H1*C*···Cl3^ii^	0.89	2.85	3.418 (4)	123
N1—H1*A*···Cl3^iii^	0.89	2.53	3.329 (4)	151
N1—H1*B*···Cl2	0.89	2.54	3.371 (6)	157
N1—H1*B*···Cl1	0.89	2.94	3.515 (4)	124
N1—H1*A*···O1	0.89	2.34	2.621 (5)	98

Symmetry codes: (i) −*x*+1, *y*−1/2, −*z*+1/2; (ii) −*x*+2, *y*−1/2, −*z*+1/2; (iii) *x*−1, *y*, *z*.
